# Mutational and structural analyses of UdgX: insights into the active site pocket architecture and its evolution

**DOI:** 10.1093/nar/gkad486

**Published:** 2023-06-07

**Authors:** Shashanka Aroli, Eui-Jeon Woo, Balasubramanian Gopal, Umesh Varshney

**Affiliations:** Department of Microbiology and Cell Biology, Indian Institute of Science, Bangalore 560012, India; Korea Research Institute of Bioscience and Biotechnology 125 Gwahak-ro,Yuseong-gu, Daejeon 34141, Republic of Korea; Department of life science, UST-KRIBB School, University of Science & Technology, Daejeon 34113, Korea; Molecular Biophysics Unit, Indian Institute of Science, Bangalore 560012, India; Department of Microbiology and Cell Biology, Indian Institute of Science, Bangalore 560012, India; Jawaharlal Nehru Centre for Advanced Scientific Research, Jakkur, Bangalore 560064, India

## Abstract

UdgX excises uracil from uracil-containing DNA to concurrently form a covalent bond with the resulting AP-DNA. Structurally, UdgX is highly similar to family-4 UDGs (F4-UDGs). However, UdgX is unique in possessing a flexible R-loop (_105_KRRIH_109_). Among the class-defining motifs, while its motif A (_51_GEQPG_55_) diverged to possess Q53 in place of A53/G53 in F4-UDGs, motif B [_178_HPS(S/A)(L/V)(L/V)R_184_] has remained unchanged. Previously, we proposed an S_N_1 mechanism resulting in a covalent bond between H109 and AP-DNA. In this study, we investigated several single/double mutants of UdgX. The H109A, H109S, H109G, H109Q, H109C and H109K mutants gain conventional UDG activity to varying levels. The crystal structures of UdgX mutants show topological changes in their active sites, rationalizing their UDG activities. The E52Q, E52N and E52A mutants reveal that E52 forms a catalytic dyad with H109 to enhance its nucleophilicity. The Q53A mutant supports that UdgX specific evolution of Q53 occurred essentially to stabilize the R-loop conformation. The R184A mutation (motif B) supports the role of R184 in substrate-binding. Taken together, the structural, bioinformatics, and mutational studies suggest that UdgX diverged from F4-UDGs, and the emergence of the characteristic R-loop in UdgX is functionally assisted by A53/G53 to Q53 changes in motif A.

## Graphical Abstract

**Graphical Abstract:**
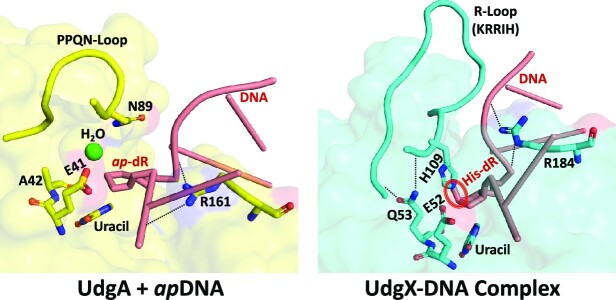

## INTRODUCTION

Uracils in DNA arise either because of cytosine (C) deamination leading to the generation of G:U wobble pairs, or due to incorporation of dUMP (U) against adenine (A) by DNA polymerase during replication (in A:U pairs). Deamination of C in DNA is facilitated by the presence of reactive oxygen species (ROS) or reactive nitrogen intermediates (RNI) (1,2). The uracils in the A:U pairs may interfere with DNA protein interactions, and if not repaired prior to genome replication, the G:U pairs lead to C to T mutations (3). Thus, organisms possess a ubiquitous enzyme, uracil DNA glycosylase (UDG), which excises uracils from DNA to initiate the base (uracil) excision repair pathway (4). As the first enzyme in the pathway, UDG hydrolyses the N-glycosidic bond between C1′ of the deoxyribose sugar and the N1 position of the attached uracil, resulting in excision of the uracil to form an apyrimidinic (AP) site in the DNA. Subsequently, in one of the repair pathways typified by *Escherichia coli*, the AP sites so generated are incised by AP-endonuclease activity (exonuclease III or endonuclease IV) to generate a 5′ end with abasic sugar (deoxyribosephosphate, dRp) at the nick. For further repair, the 5′ dRp is removed by the RecJ, and the single nucleotide gap so generated is repaired by the activities of DNA polymerase I, and DNA ligase enzymes (5–7).

The presence of characteristic short-sequence motifs in UDGs allows them to be classified into six families (8). Family 1 (Ung) enzymes are highly conserved and catalytically active on uracil-containing single-stranded (ss) or double-stranded (ds) DNAs. Ung proteins possess motif A (GQDPY), harbouring the active site residue, and motif B (HPSPLS), which forms the DNA intercalation loop important in stabilizing [ES] complex (9–13). Ung possesses exquisite specificity for uracils in DNA. Structural analyses of family 1 UDGs with uracil-containing DNA showed localisation of the side chain of L (motif B) in the DNA double helix with the target uridine flipped out into the active site pocket of the enzyme. We showed that L191 in motif B (*E. coli* Ung) stabilises [ES] complex by retaining the uridine into the flipped out conformation (14). Family 2 UDGs (Mug/TDG) have GINPG and MPSSSAR sequences as motifs A and B, respectively. Studies on *Methanobacterium thermoautotrophicum* TDG and *E. coli* Mug have shown that they excise uracils from G:U and I:U pairs in dsDNA (15). In addition, human TDG excises thymine from G:T pairs with low efficiency (16). The family 3 UDGs (SMUG) which excise uracils from ssDNA with lower efficiency than from dsDNA (17) are defined by GMNPG and HPSPRN sequences as their motifs A and B, respectively. Family 4 UDGs (UdgA) and family 5 UDGs (UdgB) possess 4Fe–4S cluster (18–21). The motifs A and B in UdgA are represented by GE(A/G)PG and HPAAVL, respectively; whereas in UdgB these motifs are represented by GLAPA and HPSPLN, respectively. While family 4 UDGs act on ssDNA and dsDNAs, the family 5 UDGs are specific for dsDNAs. Family 6 UDGs are more specific for hypoxanthine (22). All UDGs possess a conserved α/β fold suggesting a common origin of their evolution (23).

In 2015, we discovered a novel 4Fe–4S cluster containing UDG (UdgX) in *Mycobacterium smegmatis* (24). It harbours GEQPG and HPSSLLR as motifs A and B, respectively. However, unlike other UDGs, UdgX possesses an active site residue (H109) in a distinct KRRIH sequence (referred to as a ‘signature loop’ or ‘R-loop’). We subsequently demonstrated UdgX activity in *M. avium*, *Rhodococcus imtechensis* (24), and *Bradyrhizobium diazoefficiens* (25). Multiple sequence alignments of UdgX from different organisms show that motif A [GEQPG] and motif B [HPS(S/A)(L/V)(L/V)R] are conserved. However, the _105_KRRIH_109_ sequence in the R-loop is a crucial element of UdgX across different organisms. UdgX is similar to family 4 UDGs in its overall structure except for the presence of the R-loop (26,27). Our earlier studies (26) allowed us to propose a reaction mechanism of UdgX, which involves the formation of a covalent bond between H109 of UdgX with the C1′ position of the target deoxyribose sugar concurrently with the uracil excision from this site in DNA.

In this investigation, we generated a series of UdgX mutants to further our understanding of the structure function relationship of UdgX using biochemical assays, aided by X-ray crystallography and bioinformatics. These studies allow for a better understanding of the reaction mechanism of UdgX and permit us to propose that motif A (GEQPG) and R-loop sequences of UdgX co-evolved from family 4 UDGs for its specialized reaction mechanism.

## MATERIALS AND METHODS

### Clonings, DNA oligomers and growth conditions

*E. coli* TG1 and the plasmid pJET 1.2 (ThermoFisher Scientific) were used for cloning. *E. coli* Rosetta and pET14b were used for protein expression. *E. coli* strains were grown in Luria-Bertani broth (LB) or LB agar (Difco, USA). Media was supplemented with 100 μg/ml ampicillin (Amp) and 30 μg/ml chloramphenicol (Cm) as required. The enzymes used for routine cloning, PCR and DNA labeling were purchased from New England Biolabs (NEB) and Thermo Fisher Scientific, and DNA oligomers were obtained from Sigma, IDT (India) or Macrogen (South Korea).

### Recombinant plasmids

pET14bUdgX harbouring a 6× His tag at the N terminus of *Msm*UdgX was used for its expression and purification. The *Msm*UdgX mutants E52Q, E52N, E52A, Q53A, H109A, H109S, H109G, H109Q, H109C, H109K and R184A were generated by site‐directed mutagenesis (SDM) using pET14b*Msm*UdgX as template. Table S1 lists the details of available constructs and primers used for new SDMs. In brief, PCR for SDM was set up with 50 ng template DNA, 10 pmol each of the relevant forward and reverse primers, 250 μM dNTP, 1 × Q5 polymerase buffer and Q5 polymerase (NEB). PCRs included heating at 98°C for 1 min, followed by 18 cycles of denaturation at 98°C for 30 s, primer annealing at 50°C for 30 s, and extension at 72°C for 2 min. The amplicons were digested with DpnI and transformed into *E. coli* TG1. H109S/E52N, H109S/Q53A, and H109S/R184 double mutants were generated using pET14b*Msm*UdgX H109S plasmid as a template for SDM using primers specific to the second set of mutations. All plasmids were confirmed by DNA sequencing (Macrogen, South Korea).

### Purification of UdgX mutants

pET14b*Msm*UdgX and its mutants containing N-terminal 6× His tag were introduced into *E. coli* Rosetta (DE3) by transformation. Isolated colonies were inoculated into 15 ml LB containing Amp and Cm and grown until saturation (or overnight). Inoculum (1%) was added to 1.2 L LB containing Amp and Cm, grown at 37°C to an OD_600_ of 0.6 under shaking, supplemented with 0.5 mM IPTG and 0.01% FeCl_3_, and grown further for 4 h. Cells were harvested by centrifugation at 4°C, resuspended in 10 ml buffer A [20 mM Tris–HCl pH 8, 500 mM NaCl, 10% glycerol (v/v), 2 mM β-mercaptoethanol, 1 mM PMSF, and 20 mM imidazole], lysed by sonication, and centrifuged at 4°C in a pre-cooled centrifuge at 96000 rcf for 2 h. The supernatant was loaded onto a 1 ml Ni-NTA column equilibrated with buffer A, washed with 20 ml buffer A and eluted with a gradient of imidazole (20–1000 mM) in the same buffer. The fractions were analysed on 15% SDS-PAGE. Fractions enriched for UdgX were pooled, loaded onto a Superdex-75 gel filtration column, and eluted in buffer B [20 mM Tris–HCl pH 8, 400 mM NaCl, 10% glycerol (v/v) and 2 mM 2-mercaptoethanol]. The purity of UdgX was checked on 15% SDS-PAGE. Fractions with apparent homogeneity were pooled, concentrated using a 10 kDa cut-off Centricon (Millipore), and estimated by Bradford's method using bovine serum albumin (BSA) as standard. The proteins were dialyzed against buffer A containing 50% glycerol (v/v) and stored at –20°C.

### Radiolabelling of substrates by polynucleotide kinase

DNA oligomers (10 pmol) were 5′ ^32^P-end labelled (28) using 10 μCi of [^32^P] ATP (6000 Ci/mmol) and 3 units of T4 polynucleotide kinase (ThermoFisher Scientific) and purified on Sephadex G-50 minicolumns. ssU9, with a U residue at the 9th position from the 5′-end, was used as the ssDNA substrate.

### UdgX assays

*Msm*UdgX assays were carried out using the ssU9 as substrate in 10 μl reactions with 1× UDG buffer (50 mM Tris–HCl pH 8, 1 mM Na_2_EDTA, 1 mM DTT, 25 μg/ml BSA) at 37 °C for 20 min. An amount of 125 ng (5 pmol) of *Msm*UdgX or its mutants were used for the assays. The reactions were stopped by the addition of 5 μl 0.2 N NaOH, heated at 90°C for 10 min, mixed with 15 μl formamide dye (80% formamide, 0.05% each of bromophenol blue and xylene cyanol FF, 10 mM NaOH, 2 mM Na_2_EDTA), boiled for 10 min, and 15 μl aliquots were analysed on 15% polyacrylamide (19:1) 8 M urea gels. The gels were exposed to a phosphorimager cassette to acquire the images. The signals were quantified using Fujifilm Multi Gauge V2.3, and the results were plotted using GraphPad Prism 8.

### UDG kinetics assays and analysis

UDG assays to determine the kinetics of uracil excision by *Msm*UdgX mutants were performed as follows. Briefly, 0.1 pmol of ^32^P labelled ssU9 was mixed with varying amounts (0.5, 1.0, 2.0, 4.0, 8.0, 12.0, 16.0, 20.0, 25.0 and 30.0 pmol) of unlabelled ssU9 to reach the final substrate concentrations in 10 μl reactions. The reaction rate (v) was calculated from the slope of the straight line obtained by plotting product formed (Y-axis) at 2, 4, 6 and 8 min (X-axis). Concentrations of the substrate and product (nM) or their reciprocals were used to generate the Michaelis-Menten graph or Lineweaver–Burk plots (29).

### Crystallization and data collection

The initial crystallization conditions were identified using crystallization screening kits from Hampton in a 96-well sitting-drop screening format at 22°C. Crystals of the apo forms of *Msm*UdgX and its mutants were grown from solutions of each protein (15 mg ml^−1^) and the screening solution of SaltRx 18 (Hampton, cat. no. HR2-107) (2.0 M ammonium citrate tribasic (pH 7.0 and 0.1 M Bis–Tris propane pH 7.0) in a 1:1 ratio. For the crystals of the complexes of *Msm*UdgX (or its mutants) with DNA, the UdgX proteins (15 mg ml^−1^) were mixed with the 5-mer ssDNA (TTUTT) dissolved in a buffer containing 500 mM NaCl, 20 mM Tris–HCl pH 8.0, and 5 mM β-mercaptoethanol in a 1:5 molar ratio (protein:DNA) and incubated at 22°C for 15 min. The crystals of the UdgX–DNA complexes were grown from the mixtures of the protein and DNA in the mother liquor solution of SaltRx 18 in 1:1 ratio. The crystals were then transferred to a loop and flash-cooled to 100 K. No additional cryoprotectant was used prior to data collection. Diffraction data were collected at 100 K using a MAR Research image-plate system (diameter 345 mm) with Osmic mirrors and a Bruker AXS MICROSTAR ULTRA II rotating-anode X-ray generator. Intensity data were processed and scaled using MOSFLM and AIMLESS from the CCP4 program package (‘The CCP4 Suite: Programs for Protein Crystallography,’ 1994).

### Structure solution and refinement

The structures were determined by the molecular replacement method using PHASER with the structure of *Msm*UdgX H109S (PDB accession 6AJS) as a search model. Model building and structure refinement were performed using REFMAC5, COOT and PHENIX programs (30–33). From the beginning of the refinement, 5% of the total reflections were set aside to monitor the *R*_free_ value. All molecular models were generated using PyMOL or Chimera program (34,35). Ramachandran statistics were analysed using MolProbity and summarized along with the crystallographic data statistics (Tables S2A–2C).

### Mutual information analysis and identification of coevolving residues

The coevolution of amino acid residues was determined using CoeViz (a web-based tool for coevolution analysis of protein residues) using *Msm*UdgX as the query sample (36). The results were visualized using tools incorporated into the CoeViz web server. The server computes pairwise coevolution scores using three metrics: mutual information, chi-square statistics, and Pearson correlation. In addition, an option for computing conservation scores based on Joint Shannon Entropy is provided. The tool is part of the POLYVIEW-2D protein structure visualization server and is available from the resulting pages of POLYVIEW-2D (36).

### Sequence and structural analysis of UdgX evolution

To understand the structural and evolutionary changes that caused the divergence of UdgX from family 4 UDGs, a detailed primary and tertiary structural comparison of UdgX was performed by taking family 4 UDGs as a reference. In brief, about 5000 sequences were selected for the primary sequence analysis based on our sequence search by taking *Msm*UdgX as a query sequence. The sequence files were then aligned using the ClustalW algorithm incorporated in MEGA-X with default parameters, without any gaps (37), and the output file was used for sequence conservation and coevolution analysis. A python script was written to identify the most abundant residue in each position of the primary structure in the multiple sequence alignment (MSA) file. R107 and H109 positions of *Msm*UdgX are the most conserved and unique to UdgX. The MSA file was sorted for the presence of R107 and H109 (corresponding position) to filter all the UdgX sequences from family 4 UDGs. The filtered sequences were further analysed to select the residues with >95% conservation in their respective positions. The identified residues were further compared with the family 4 UDGs in the corresponding positions to filter them out as unique to UdgX. The conserved residues unique to UdgX were considered to understand their role in structure and evolution of UdgX.

## RESULTS

### Rationale for *Msm*UdgX activity on uracil-containing DNA

**Figure 1.**
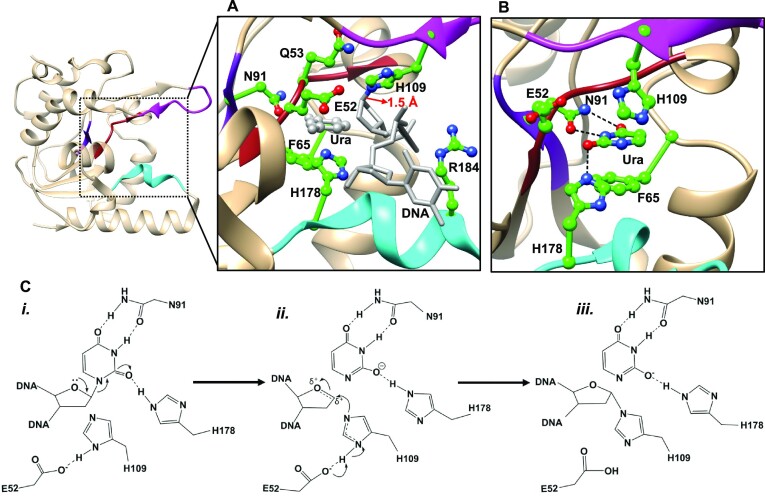
Structure based mechanism of *Msm*UdgX action on uracil containing DNA. (**A**) Critical components of *Msm*UdgX function. The residues containing motif A (brown), motif B (cyan), a sequence harboring N91 (purple), and the R-Loop (magenta) are highlighted. Critical residues contributing to the active site of *Msm*UdgX are color coded with atomic colors, and the DNA and uracil are color coded with grey. The covalent bond between H109 and C1′ of AP-DNA (1.5 Å) is seen (PDB ID: 6AJO). (**B**) Uracil bound in the active site of *Msm*UdgX, post its cleavage from the DNA backbone. The hydrogen bonding interactions between uracil and UdgX are shown in dashed lines (PDB ID: 8IIE). (**C**) Discrete steps in the reaction mechanism of *Msm*UdgX. The flow of electrons is depicted with curved arrows.

UdgX is structurally similar to the family 4 UDGs (24,26,27). It possesses α/β/α fold with four central parallel β stranded sheet sandwiched between the α helices. The functionally important elements such as motif A, motif B, and the R-Loop sequences of *Msm*UdgX, and the conserved sequence _90_TNAV_93_ found in UDGs are shown in Figures 1A and B. UdgX follows a unimolecular substitution (S_N_1) reaction mechanism (Figure 1C), wherein the glycosidic bond between uracil and the deoxyribose sugar in the DNA is replaced by a unique His-deoxyribose sugar covalent bond (1.5 Å) in UdgX–DNA upon excision of uracil (Figure 1A and C) (26). Briefly, as a first step in the reaction, uracilate anion formation is facilitated by hydrogen bonding of H178 and N91 with the uracil in the active site pocket, resulting in a ‘pulling’ force on uracil and weakening of the glycosidic bond. This step of UdgX action is like the other UDGs. In the next step, E52 (motif A) activates the H109 (R-Loop), converting it into an efficient nucleophile to facilitate its covalent bonding with C1′ (having a positive charge imparted upon it by departure of uracilate anion) of the target deoxyribose sugar (26). In canonical UDGs, the AP-deoxyribose sugar cation is stabilized by a hydroxylate anion generated by the splitting of a water molecule (38). Indeed, in the H109S mutant, which converts UdgX into a conventional UDG with turnover for uracil excision (24), the crystal structure revealed that S109 enables the localisation of a water molecule at this position (26). However, the basis for differential uracil excision activities by H109 substitutions with other amino acids remained unclear. Furthermore, based on our structural analyses, we suggested that R184 in the DNA intercalation loop (motif B) facilitates localization of the target U in the active site. Further, Q53 was proposed to play a role in appropriately orienting H109 (27). The latter study also surmised that Q53 may play a role in activating a nucleophile resulting in the formation of a covalent bond between H109 and DNA. Overall, the side chains of E52, Q53, N91, H109, H178 and R184 were proposed to play crucial roles in UdgX activity. To better understand the reaction mechanism of UdgX, we evaluated the biochemical and structural features of single/double mutants at H109, E52, Q53 and R184.

### *Msm*UdgX H109 mutants show changes in their catalytic activities

Substitutions of His109 in *Msm*UdgX with Ser, Gly, Ala, Gln, Cys and Lys showed the loss of its covalent complex formation with DNA but gained canonical UDG activities (Supplementary Figure S1A). In the reactions of the mutant proteins with ssU9, the entire substrate was converted to product by the H109G mutant, while hardly any substrate was utilized by the H109K mutant (Table 1, Figure S1B). We note that smaller and hydrophilic amino acid substitutions at His109, such as Gly or Ser showed higher *K*_cat_/*K*_m_ values. However, H109 substitutions by Gln or Lys, showed poor activity. Both Cys and Ala mutations of H109 showed intermediate *K*_cat_/*K*_m_ values. These changes in the catalytic activities possibly reflect steric hindrance by large side-chain substitutions of H109.

**Table 1.** Kinetics parameters of *Msm*UdgX mutants. *V*_max,_*K*_m_ and the errors in *V*_max_ and *K*_m_ were calculated from the Lineweaver–Burk (LB) plot. *K*_cat_ was calculated by dividing *V*_max_ with [Et] (enzyme total = 200 nM)

Protein	*V*_max_ (nmol l^−1^min^−1^)	*K*_m_ (nM)	*K*_cat_ (min^−1^)	*K*_cat_/*K*_m_ (×10^−3^) (min^−1^nM^−1^)
**WT**	-	-	-	-
**H109K**	0.67 ± 0.15	1002.20 ± 907.41	0.003 ± 0.001	0.001 ± 0.000
**H109Q**	7.16 ± 0.39	292.10 ± 14.79	0.036 ± 0.002	0.123 ± 0.003
**H109G**	22.97 ± 2.31	174.76 ± 29.09	0.115 ± 0.012	0.671 ± 0.041
**H109S**	11.57 ± 1.42	154.92 ± 21.29	0.058 ± 0.007	0.382 ± 0.052
**H109C**	12.48 ± 2.06	228.11 ± 50.79	0.062 ± 0.010	0.288 ± 0.041
**H109A**	4.83 ± 0.35	184.50 ± 13.52	0.024 ± 0.002	0.131 ± 0.001
**H109S/E52N**	2.21 ± 0.20	1063.17 ± 121.32	0.006 ± 0.004	0.005 ± 0.001
**H109S/Q53A**	28.27 ± 2.44	176.12 ± 19.25	0.141 ± 0.012	0.806 ± 0.019
**H109S/R184A**	6.15 ± 0.31	308.05 ± 25.82	0.031 ± 0.002	0.101 ± 0.011

**Figure 2.**
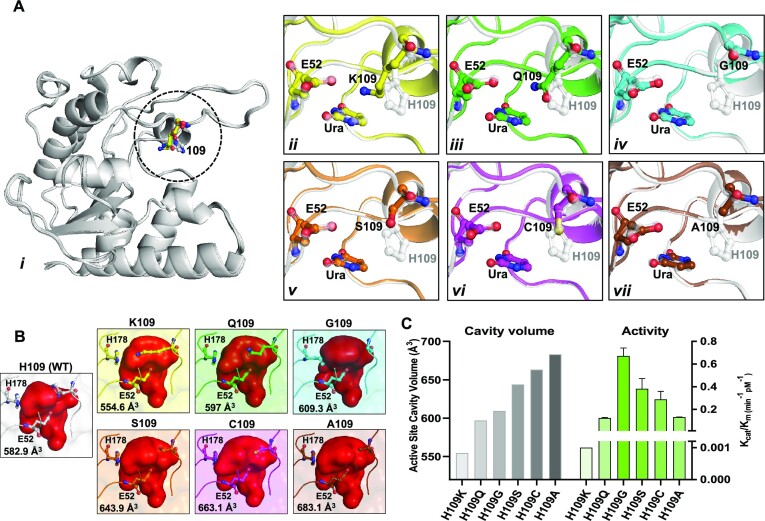
*Msm*UdgX H109 mutants show changes in their catalytic activity.(**A**) Structural rationale for the effect of H109 mutations on catalytic site. Crystal structures of H109K (yellow, (PDB ID: 8IIN), H109Q (green, PDB ID: 8IIP), H109G (cyan, PDB ID: 8IIL), H109S/R184A (orange, PDB ID: 8IIT), H109C (magenta, PDB ID: 8III) and H109A (brown, PDB ID: 8IIG), mutants with uracil in the active sites cavity are superposed with WT *Msm*UdgX (white, PDB ID:8IIE) structure with uracil in its active site. The E52, the 109 mutant positions, and uracil are shown in ball and stick model. (**B**) Active site cavities of individual mutants of *Msm*UdgX. Active site volume was calculated using Hollow program, and the same program was used for generating corresponding figures. (**C**) Bar graph representing the *Msm*UdgX H109 mutants active site cavity volume (left y-axis), and their respective *K*_cat_/*K*_m_ values (right y-axis). *K*_cat_ and *K*_m_ values were calculated from double reciprocal plot (Lineweaver–Burk plot) and the values were plotted on *xy* graph.

However, to better understand the mechanism of uracil excision by the H109 mutants, we determined their structures along with a pentameric DNA, TTUTT by X-ray crystallography. Data collection and refinement statistics are provided in Tables S2A–S2C. The DNA backbone could not be modelled into the experimental electron density map in any of the mutant structures that were determined. Nonetheless, all structures revealed the excised uracil and the side chain substitutions at position 109 (Figures S2A and S2B). The structures also revealed invariant interactions of N91 and H178 with uracil (Figures S2C and S2D). Further, the overlay of all the structures of UdgX H109 mutants (Figure 2A, panel i) showed that the overall structures are very well conserved with a Cα RMSD of *ca* 0.2 Å. The superpositions of the various mutants at position 109 with the wild type UdgX (white) are shown in Figure 2A (panels ii-vii). As anticipated, we note a clear difference in the sizes of the active site cavities in the various mutants. The active site volumes of the H109 mutants were calculated using the Hollow program (39). The active site cavity is mainly formed by motif A, motif B, and the R-loop at the edge of the four parallelly placed β strands. This defines the space available for the ligand molecules to position in the active site cavity (Figure 2B, and Table S3). When we arranged the mutants in the order of increasing cavity sizes (Figure 2C, left), we noted that the canonical UDG activity of the mutants increased with their increasing cavity sizes to begin with and then with a further increase in the cavity size, the UDG activity declined (Figure 2C, right). It suggested that the initial increase in UDG activity (H109K to H109Q to H109G) occurred because of the efficiency with which the water molecule could be localized to make a nucleophilic attack on the C1′ position. However, a further increase in the cavity size (H109S to H109C to H109A) perhaps destabilizes the bound water, thereby decreasing the UDG activity. Interestingly, G109 the smallest amino acid substitution of H109 showed lower cavity volume than Ser, Cys or Ala due to an overall structural change near the active site.

### E52 is essential to activate H109 for UdgX activity

**Figure 3.**
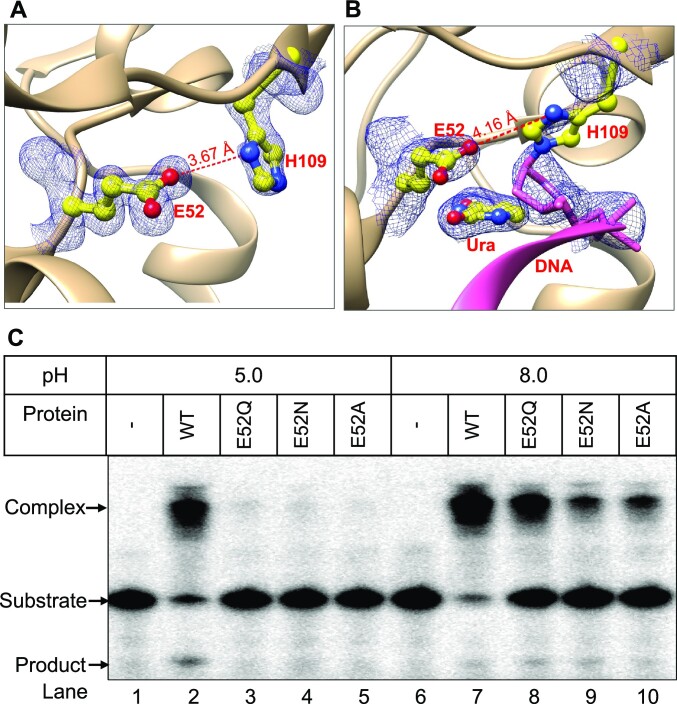
E52 is essential to activate H109 for UdgX activity.(**A**) Re-refined structure of *Msm*UdgX showing the distance between OE2 of E52 and ND1 of H109 (PDB ID: 6AIL). E52 and H109 are color coded with the atomic colors. The (*2mF_o_– DF_c_*) map is contoured at 0.5 σ and presented as blue mesh. (**B**) Re-refined structure of *Msm*UdgX in complex with uracil containing DNA indicating the distance between OE2 of E52 and ND1 of H109. E52, H109 and uracil are color coded with atomic colors and DNA is color-coded with light pink. The (*2mF_o_ – DF_c_*) map is contoured at 0.5 σ and presented as blue mesh (PDB ID: 6AJO). (**C**) Activity assay of *Msm*UdgX mutants at pH 5.0 and pH 8.0. Activity assays for the mutants were performed at pH 5.0 and pH 8.0, using ssU9 substrates labelled with ^32^P at the 5′ end. The reaction was conducted for 20 min, resolved on 8 M urea PAGE and analysed by phosphorimaging.

E52 is conserved among family 4 UDGs and in UdgX. Based on our previous structural analysis, we had proposed that E52 participates in a catalytic dyad with H109 to activate it for a nucleophilic attack onto C1′ position of the target the deoxyribose sugar cation (26). Further, in the structure of H109S mutant, a water molecule was appropriately located near S109 for activation by E52 (26). In previous structure determinations (6AIL) (26) and (6IO9) (27) of *Msm*UdgX, the OE2 of E52 is positioned at distances of 4.34 and 5.53 Å, respectively from the ND1 of H109. However, such distances are unfavourable to activate H109. Thus, we carried out further refinements of the structure. This resulted in a new model where the distance between OE2 of E52 and ND1 of H109 is 3.67 Å, and compatible with the withdrawal of proton by E52 from ND1 of H109 (Figure 3A). In the revised positioning, H109 retains the same rotameric form in both the DNA unbound and bound forms (Figure S3A). However, in the DNA bound form, the distance between E52 and H109 is 4.16 Å to allow covalent bond (1.5 Å) formation of H109 with the C1′ of the AP-sugar (Figure 3B).

To further validate the role of E52, we combined the H109S mutation (where a water molecule is allowed to locate in place of His ring) with the E52N mutation and assessed its effect on the uracil excision activity. Expectedly, the uracil excision activity of the double mutant (H109S/E52N) was lowered compared to the H109S mutant (Figure S3C). The *K*_cat_ of the double mutant (H109S/E52N) decreased by ∼10-fold as compared to the H109S (Table 1). The *K*_m_ of the double mutant increased by ∼7-fold, resulting in an overall reduction of ∼75-fold in *K*_cat_/*K*_m_ (Table 1, and Figure S3D). Consistent with these observations, the distance of 4.71 Å between OG of S109 and OE2 of E52 in the H109S mutant increased to 5.71 Å between OG of S109 and ND2 of N52 in the H109S/E52N mutant (Figure S3E and F).

To further support the role of E52 in the withdrawal of a proton from H109 (Figure 1C, step ii), we reasoned that at acidic pH, the nucleophilic properties of H109 in UdgX (wild type) would be compromised. Under these conditions, activation of H109 would become critically dependent on proton withdrawal by E52. Thus, the mutations at E52 that dampen its proton withdrawal activity, would be expected to decrease the covalent bond formation activity of UdgX, particularly at a lower pH. As shown in Figure 3C, the complex formation activities of E52Q, E52N and E52A (lanes 8–10) were compromised at pH 8.0, as compared wild type control (lane 7) and severely compromised to an undetectable level at pH 5.0 (Figure 3C, lane 3 to 5). An efficient complex formation by the wild type UdgX at pH 5.0 (lane 2) ruled out the loss of structural integrity of UdgX at the lower pH of 5.0. Not unexpectedly, in the same reaction, we see a band corresponding to uracil excision (followed by cleavage of the backbone) in the substrate (i. e. following uracil excision but lacking nucleophilic attack by H109) (Figure 3C, lane 2 and Figure S3G). These observations emphasise the role of E52 in the catalytic dyad with H109 (Figure 1C, step ii). Any delays in the nucleophilic attack onto the C1′ position by H109 allows the release of the AP DNA from UdgX, which is, in turn, seen as a cleaved product under the reaction conditions. Taken together, these observations explain the mechanism of covalent bond formation of UdgX with the DNA through H109.

### Q53 is an enabling change in the evolution of *Msm*UdgX

**Figure 4.**
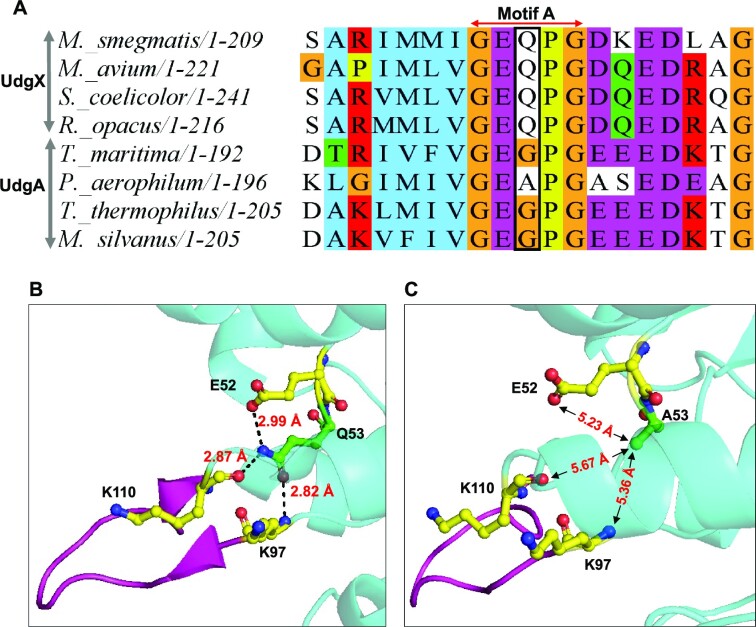
Q53 is an enabling change in the evolution of *Msm*UdgX.(**A**) Multiple sequence Alignment of motif A sequence of UdgX and Family 4 UDGs (UdgA) showing the divergence from G/A in UdgA to Q in UdgX. (**B**) Crystal structure showing the interaction between K97, Q53 and K110 (PDB ID 6AIL). (**C**) Crystal structure showing the interactions between K97, A53 and K110 in H109S/Q53A double mutant (PDB ID: 8IIR).

The motif A sequences of UdgX and family 4 UDGs are represented by GEQPG and GE(A/G)PG, respectively (Figure 4A). Q53 is highly conserved in UdgX. It is positioned in the active site pocket, and it is also the main difference between motif A of UdgX and the remaining family 4 UDGs. Not surprisingly, Q53 was surmised to activate H109 (27). However, to understand the precise role of Q53 in UdgX, we re-analysed the *Msm*UdgX structure with a focus on Q53. Q53 makes hydrogen bonds with the O of K110 (2.87 Å) and N of K97 (2.82 Å) (Figure 4B, and Figure S4A). These interactions remain intact in the DNA bound form with bond distances of 2.69 and 2.79 Å, respectively (Figure S4B and C). Thus, these interactions may help anchor the R-loop towards the active site of UdgX. In addition, Q53 makes a weaker hydrogen bond with OE2 of E52 (2.99 Å). However, this bond is stretched to 3.45 Å in the DNA-bound form of UdgX (Figure S4C). To evaluate this in more detail, we generated a Q53A mutant wherein the motif A of *Msm*UdgX was changed to GEAPG to correspond to the motif A of family 4 UDGs. The time course assay did not reveal a difference in the ability of the Q53A mutant in complex formation (Figure S4D). Subsequently, we incorporated the Q53A mutation with the H109S mutant to investigate its role in a surrogate assay of uracil excision (with turnover). The Q53A mutation in the H109S mutant increased its uracil excision activity (Figure S4E). Further, the kinetic parameters of uracil excision (Table 1, Figure S4F) showed an increase of >2-fold in the *K*_cat_/*K*_m_ of the double mutant. When compared with the structure of UdgX (wild type), the hydrogen bonding interactions observed between Q53 with N of K97 and O of K110 were lost in H109S/Q53A double mutant due to the absence of Gln side chain in the mutant (Figure 4C). This would likely change the positioning of the R-Loop near the active site, leading to an increase in the activity of the H109S/Q53A double mutant, compared to the H109S single mutation. Taken together, these observations suggest that the evolution of Q53 in UdgX (from A53/G53 in family 4 UDGs) disfavours uracil excision activity. This evolutionary feature could enable covalent complex formation by UdgX.

### Q53 is a divergent mutant of family 4 UDGs motif A

**Figure 5.**
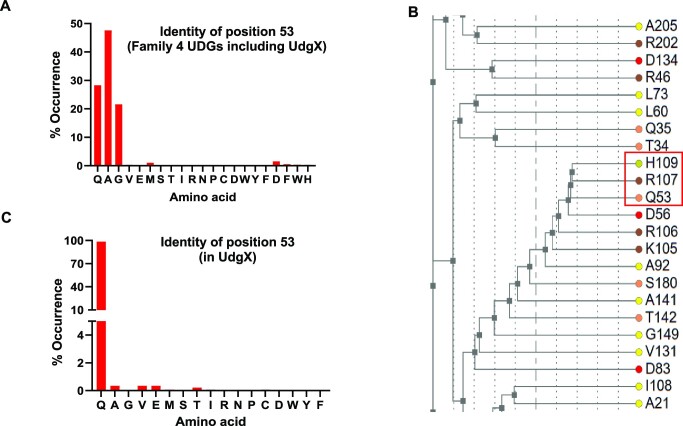
Q53 is a divergent mutant of family 4 UDGs motif A.(**A**) XY graph depicting the percentage occurrence of 53 (motif A) equivalent position occupied by various residues in family four UDGs (including UdgX). Individual sequences (5000) were aligned using the default parameters of ClustalW (without gaps) to obtain the multiple sequence alignment (MSA) file. The number of occurrences of individual residue was divided by the total number of samples to get the percent occurrence of each residue in the corresponding position. (**B**) Clustering tree generated from CoeViz server showing co-occurrence of Gln53, Arg107 and His109 in UdgX protein. (**C**) XY graph depicting the percentage occurrence of 53 (motif A) equivalent position occupied by various residues in UdgX proteins. Individual sequences with R107 and H109 residues in the MSA file of 5000 sequences were filtered-in to calculate the occurrence of each residue in the equivalent position of 53 in *Msm*UdgX. The number of occurrences of individual residue was divided by the total number of filtered samples to get the percentage occurrence of each residue in the corresponding position.

To further understand the importance of Q53 in UdgX, we compared the multiple sequence alignments of all family 4 UDGs (inclusive of UdgX). Q, A, or G are the most predominant residues at position 53 (Figure 5A). Likewise, we note that the residue 107 is R or P, and residue 109 is H or N (Figures S5A and S5B). A manual check of >1000 individual sequences revealed that all the sequences with R107 and H109 correspond to UdgX (with the presence of the entire R-loop), whereas P107 and N109 corresponded to the sequences of the typical family four UDGs (i.e. excluding UdgX). Also, in our previous study, we showed that the catalytic role of N89 in *Tth*UDG is carried out by H109 in *Msm*UdgX (25). This enabled us to conclude that R107 and H109 are the signature sequences of the UdgX. Further, to establish a correlation between Q53 and the R-loop residues, we performed an analysis using the CoeViz web server (35,39). The emergence of Q53 is closely related to the R-Loop residues in their occurrence (Figure 5B and Figure S5C). Based on this coevolution analysis, we suggest that Q53 in UdgX is a result of divergence from A/G at this position in motif A of the typical family 4 UDGs. The co-occurrence of Q53 vis-a-vis the two highly conserved residues of R107 and H109 in UdgX proteins is >98% (Figure 5C, Figure S5D, and Figure S5E). These observations suggest that the R-loop of UdgX evolved from the flexible loop of the typical family 4 UDGs, together with the emergence of Q53 (from A53 or G53) in motif A.

### R184 is a key mediator of substrate recruitment in UdgX

**Figure 6.**
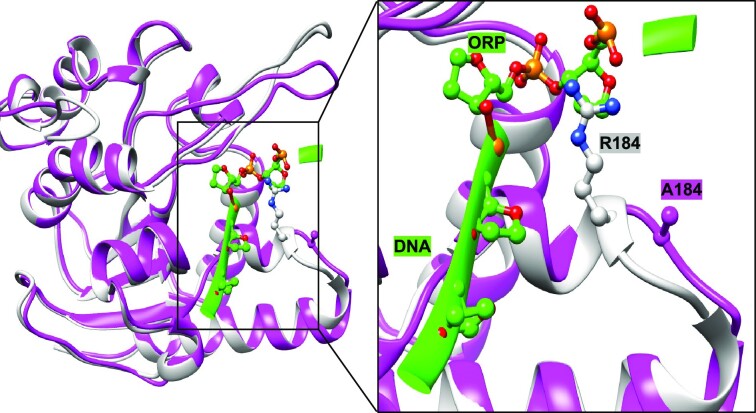
R184 is a key mediator of substrate recruitment in *Msm*UdgX. Structural rationale for the effect of R184 mutation. Superposition of *Msm*UdgX WT (white) with DNA (green) bound form (PDB ID: 6AJO) and R184A mutation (magenta, PDB ID: 8IIT) shows the lack of interaction between DNA backbone and R184A in the mutant (ORP corresponds to deoxyribonucleoside phosphate).

From the crystal structures (26,27), we observed that R184, P68, A69, S180, H178, A141, S181, V154, T155 and A153 help in the stabilisation of the enzyme substrate complex by making various interactions with the DNA backbone. R184 in motif B, in particular, changes its position in DNA bound form (compared with its apo form). Also, R184 makes interaction with the DNA backbone (P + 1) (Figure 6A). R184 is conserved both in UdgX and family 4 UDGs. We combined H109S and R184A mutations and measured the kinetics of uracil excision (Figure S6B and Table 1). H109S/R184A mutant showed nearly 2-fold increase in its *K*_m_ as compared to H109S, and about 4-fold decrease in *K*_cat_/*K*_m_ (Table 1). To understand this further, we determined the structure of H109S/R184A mutant (Table S2C) and compared the R184A region of it with the structure of UdgX–DNA complex (Figures S6D and S6E). The NG and NE of R184 established H-bonds of 3.53 and 3.55 Å lengths with the phosphate backbone of DNA (*P* + 1), and the NE of R184 makes another hydrogen bond with (*P* – 1) of DNA backbone with 2.32 Å distance. These observations emphasise the role of R184 in binding of DNA substrate with UdgX.

## DISCUSSION

Of all the UDGs, UdgX is novel in its mechanism of action. Unlike other UDGs, which excise uracil with a turnover, UdgX excises uracil from DNA only to capture it by concomitantly forming a covalent bond (through H109) with the C1′ position of the abasic deoxyribose sugar generated upon cleavage of the *N*-glycosidic bond (26,27). We proposed that an important physiological role UdgX might play is to protect the genome from extensive fragmentation by protecting the AP-sites, which would otherwise be generated by the action of other UDGs in the cell, especially under the stress conditions that promote the conversion of C to U. It may well be that some of the physiological changes in the cell may also lead to a greater incorporation of uracils in DNA (2). The UdgX–DNA complexes generated by the action of UdgX on uracil sites in DNA may then be resolved by the action of RecA dependent pathways (24) or by the action of proteases (40). While the precise physiological role of UdgX remains largely unknown, based on its exquisite specificity for uracils in DNA, UdgX has been exploited in various genome wide methodologies (41,42). Thus, a better understanding of the mechanistic details of the UdgX chemistry on DNA, and the evolution of its active site pocket would help in its better utilization in the genome technologies, expanding its utility on even the non-uracil sites in DNA.

In this investigation, we advanced our earlier knowledge on the architecture and evolution of the active site pocket of the UdgX. Structurally, UdgX is closest to the family 4 UDGs (24,26). However, it differs from the family 4 UDGs in its altered motif A (_51_GEQPG_55_), and in possessing the R-loop (_105_KRRIH_109_). As we show in this study, the role of E52 in UdgX is to constitute a catalytic dyad with H109 of the R-loop and contribute to enhance the nucleophilic attack of H109 on the C1′ of the target deoxyribose. For example, at lower pH, the role of E52 becomes critical in activating H109 (Figure 3C). In addition, mutation of E52 to A52, results in undetectable nucleophilic activity of H109 in forming a covalent complex with DNA (Figure 3C). Importantly, E52 retains its role in activating the water molecule when located at this site (as in the family 4 UDGs) in the H109 mutants such as H109S, H109G, H109A, H109Q, H109C, H109K that we tested in our studies.

Another residue that is highly conserved in UdgX active site is Q53. The structural analysis shows that Q53 makes hydrogen bonds with K97 and K110 in the R-loop of UdgX (Figure 4B). We believe that such a role would facilitate in shaping the R-loop. Further, as shown by the uracil excision activity of the H109S/Q53A double mutant (Table 1), the evolution of Q53 (from A or G in family 4 UDGs), appears also to dampen the uracil excision activity. Such a loss in the catalytic activity of the family 4 UDGs might have facilitated the evolution UdgX. The Q53 was proposed to play a role in positioning H109 for a nucleophilic attack on the C1′ of the target deoxyribose sugar (27). However, the fact that the change of Q53 to A53 (family 4 like motif A) did not result in any detectable change in the complex formation, does not suggest a role for Q53 in the actual chemistry of catalysis (activating the H109) for a nucleophilic attack on the target C1′ position, as surmised (27). Also, if Q53 indeed participated in actual catalysis, we would have seen a large drop in the catalytic activity of the H109S/Q53A mutant in uracil excision (which on the contrary, increased). Importantly, Q53 (along with H109 and R107) is highly conserved in all UdgX proteins (Figure 4A and Figure 5C), and our correlation analysis shows that these residues coevolved in UdgX (Figure 5B, C and Figure S5). In fact, in our earlier study (26), we showed that R107 is one of the important residues in UdgX as it makes a salt bridge with D56 and D59 residues in the active site pocket.

The critical role of H109 in the catalytic mechanism of UdgX was identified at the time of the discovery of UdgX (24) and then validated by the structural analysis of its complex with uracil containing DNA (26,27). In this study, we characterized a series of mutations at His109. Our studies suggest a major role of H109 in carrying out the unique role of UdgX in forming a covalent bond appears to be facilitated by its ability in avoiding a water molecule in this location, whose activation by E52 would otherwise result in the release of the AP-DNA, as in other UDGs. Such a role of H109 is supported by the kinetics of uracil excision by the several mutations we tested at position 109 (Figure 2C). In fact, a comparison between the LSQ superposed structures of *Msm*UdgX with *Tth*UdgA shows that the H109 position of UdgX is occupied by a water molecule in *Tth*UdgA (26,43).

In addition, our biochemical and structural analysis of the R184 mutant shows that R184 is critical in binding to the DNA by establishing multiple interactions with the phosphate backbone in the neighbourhood of the uracil in the DNA. The R184A mutation results in ∼2-fold increase in *K*_m_ for DNA binding and an overall reduction of ∼4 fold in *K*_cat_/*K*_m_ value of the mutant confirming its role in stabilization of the enzyme substrate complex.

Finally, our observations in this study have not only consolidated the mechanism of catalysis by UdgX but also offered evidence of the evolution of the architecture of its active site pocket from the family 4 UDGs. The evolution appears to occur to perform the specialized role of UdgX in making a covalent bond with DNA by recognizing uracil residues in it and by replacing the uracil-DNA bond with the H109-DNA bond to yield an irreversible UdgX–DNA complex. We believe these studies would allow us to expand the repertoire of the bases in DNA that UdgX can act on and in increasing the scope of engineered UdgX proteins for their wider use in DNA technologies.

## Supplementary Material

gkad486_Supplemental_File

## Data Availability

The crystallographic data that support the findings of this study are available from the PDB (https://www.rcsb.org/) under the following accession codes: UdgX-uracil, 8IIE; UdgX H109A, 8IIF; UdgX H109A-uracil, 8IIG; UdgX H109C, 8IIH; UdgX H109C-uracil, 8III; UdgX H109G, 8IIJ; UdgX H109G-uracil, 8IIL; UdgX H109K, 8IIM; UdgX H109K-uracil, 8IIN; UdgX H109Q, 8IIO; UdgX H109Q-uracil, 8IIP; UdgX H109S/E52N, 8IIQ; UdgX H109S/Q53A, 8IIR; UdgX H109S/R184A, 8IIS; UdgX H109S/R184A-uracil, 8IIT. Any other data underlying this article will be shared on reasonable request to the corresponding author.
