# Supplementary material for: Mutational and structural analyses of UdgX: insights into the active site pocket architecture and its evolution
Source: Nucleic Acids Res. 2023 Jun 7;51(13):6554–65. doi: 10.1093/nar/gkad486 (PMC10359453; doi:10.1093/nar/gkad486)
Supplement: gkad486_Supplemental_File [file gkad486_supplemental_file.pdf]

# Supplementary Tables and Figures

## Supplementary Table S1

## List of strains, plasmids and oligomers

| Strain | Genotype | Reference/Source |
|---|---|---|
| *E. coli* BL21 (DE3) | F- ompT hsdSB(rB-mB-) gal dcm (DE3). | Novagen |
| *E. coli* BL21 Rosetta (DE3) | F- ompT hsdSB(rB-mB-) gal dcm (DE3) pRARE | Novagen |
| pET14bMsmUdgX | pET14b plasmid with MsmUdgX cloned in its NdeI/HindIII sites. | Sang et al., 2015 |
| pET14bMsmUdgX/H109A | pET14b plasmid with MsmUdgX H109A mutant cloned in its NdeI/HindIII sites. | Sang et al., 2015 |
| pET14bMsmUdgX/H109G | pET14b plasmid with MsmUdgX H109G mutant cloned in its NdeI/HindIII sites. | Sang et al., 2015 |
| pET14bMsmUdgX/H109Q | pET14b plasmid with MsmUdgX H109Q mutant cloned in its NdeI/HindIII sites. | Sang et al., 2015 |
| ssU9 | Substrate for UDG assay having uracil in 9th position. 5′d(CTCAAGTGUAGGCATGCAAGAGCT)3′ | Kumar et al., 1997 |
| MsmUdgX FP | 5′d(TGCATATGGCGGGTGCGCAAGAT)3′ | Sang et al., 2015 |
| MsmUdgX RP | 5′d(CAAGCTTGCAGATGGGCTCCATC)3′ | Sang et al., 2015 |
| MsmUdgX H109S FP | 5′d(GCAAACGACGCATAAGCAAGACCCCCAGT)3′ | This study |
| MsmUdgX H109S RP | 5′d(ACTGGGGGTCTTGCTTATGCGTCGTTTGC)3′ | This study |
| MsmUdgX H109K FP | 5′d(GCAAACGACGCATAAAGAAGACCCCCAGT)3′ | This study |
| MsmUdgX H109K RP | 5′d(ACTGGGGGTCTTCTTTATGCGTCGTTTGC)3′ | This study |
| MsmUdgX H109C FP | 5′d(GCAAACGACGCATATGTAAGACCCCCAGT)3′ | This study |
| MsmUdgX H109C RP | 5′d(ACTGGGGGTCTTACATATGCGTCGTTTGC)3′ | This study |
| MsmUdgX E52Q FP | 5′d(CATGATGATCGGCCAGCAGCCCGGTG)3′ | This study |
| MsmUdgX E52Q RP | 5′d(CACCGGGCTGCTGGCCGATCATCATG)3′ | This study |
| MsmUdgX E52N FP | 5′d(CATGATGATCGGCAACCAGCCCGGTG)3′ | This study |
| MsmUdgX E52N RP | 5′d(CACCGGGCTGGTTGCCGATCATCATG)3′ | This study |
| MsmUdgX E52A FP | 5′d(CATGATGATCGGCGCGCAGCCCGGTG)3′ | This study |
| MsmUdgX E52A RP | 5′d(CACCGGGCTGCGCGCCGATCATCATG)3′ | This study |
| MsmUdgX Q53A FP | 5′d(GATCGGCGAGGCGCCCGGTGACAAAG)3′ | This study |
| MsmUdgX Q53A RP | 5′d(CTTTGTCACCGGGCGCCTCGCCGATC)3′ | This study |
| MsmUdgX R184A FP | 5′d(TCGTCTCTGCTGGCCGGACCCAAGGAG)3′ | This study |
| MsmUdgX R184A RP | 5′d(CTCCTTGGGTCCGGCCAGCAGAGACGA)3′ | This study |

**Supplementary Table S2A**

**Data collection and refinement statistics of crystal structures of *Msm*UdgX mutants.**

| **Data collection statistics** | **WT-DNA** | **H109A** | **H109A-DNA** | **H109C** | **H109C-DNA** |
|---|---|---|---|---|---|
| PDB ID | 8IIE | 8IIF | 8IIG | 8IIH | 8III |
| Space group | $P2_1$ | $P2_1$ | $P2_1$ | $P2_1$ | $P2_1$ |
| a (Å) | 36.42 | 36.44 | 36.71 | 36.38 | 36.48 |
| b (Å) | 51.33 | 51.51 | 50.72 | 51.51 | 51.37 |
| c (Å) | 55.14 | 54.66 | 55.06 | 54.90 | 54.99 |
| β (°) | 104.98 | 104.62 | 104.50 | 104.67 | 104.72 |
| $V_M$ ($Å^3$ $Da^{-1}$) | 2.11 | 2.03 | 2.11 | 2.03 | 1.99 |
| Solvent content (%) | 41.67 | 39.33 | 41.76 | 39.48 | 38.33 |
| **Resolution range (Å)** | 29.02-1.67 (1.69-1.67) | 52.89-1.65 (1.68-1.65) | 29.11-2.30 (2.38-2.30) | 29.06-2.13 (2.19-2.13) | 28.12-1.72 (1.75-1.72) |
| Unique reflections | 22686 (864) | 22333 (729) | 8832 (848) | 10487 (899) | 18866 (1117) |
| Multiplicity | 4.8 (3.9) | 4.8 (3.4) | 3.8 (3.7) | 3.1 (3.0) | 3.3 (3.2) |
| Completeness (%) | 98.0 (75.1) | 94.5 (64.5) | 100.0 (100.0) | 94.2 (99.9) | 89.7 (100.0) |
| $<I/\sigma(I)>$ | 17.9 (4.6) | 14.6 (2.8) | 5.6 (2.4) | 6.8 (2.3) | 7.4 (2.1) |
| $R_{merge}$ (%) | 5.0 (28.7) | 5.7 (30.6) | 13.6 (43.2) | 9.9 (34.5) | 8.5 (51.5) |
| $CC_{1/2}$ | 0.996 (0.598) | 0.996 (0.821) | 0.982 (0.753) | 0.989 (0.824) | 0.991 (0.721) |
| **Refinement and model statistics** | | | | | |
| $R$ factor (%) | 15.83 | 16.83 | 18.58 | 17.19 | 20.11 |
| $R_{free}$ (%) | 18.79 | 20.27 | 22.22 | 22.17 | 23.25 |
| R.M.S Bond lengths (Å) | 0.017 | 0.007 | 0.003 | 0.008 | 0.008 |
| R.M.S Bond angles (°) | 1.399 | 0.931 | 0.610 | 0.973 | 1.013 |
| **Ramachandran plot statistics (residues in %)** | | | | | |
| Favoured region | 100.0 | 100.0 | 99.02 | 98.54 | 100.0 |
| Allowed region | 0.0 | 0.0 | 0.49 | 1.46 | 0.0 |
| Disallowed region | 0.0 | 0.0 | 0.49 | 0.0 | 0.0 |

**Supplementary table S2B**

**Data collection and refinement statistics of crystal structures of *Msm*UdgX mutants.**

| **Data collection statistics** | **H109G** | **H109G-DNA** | **H109K** | **H109K-DNA** | **H109Q** |
|---|---|---|---|---|---|
| PDB ID | 8IIJ | 8IIL | 8IIM | 8IIN | 8IIO |
| Space group | $P2_1$ | $P2_1$ | $P2_1$ | $P2_1$ | $P2_1$ |
| a (Å) | 36.71 | 36.78 | 36.45 | 36.46 | 36.69 |
| b (Å) | 50.58 | 50.83 | 51.75 | 51.54 | 51.14 |
| c (Å) | 54.83 | 55.42 | 54.63 | 54.86 | 54.58 |
| β (°) | 104.22 | 105.08 | 104.83 | 104.71 | 104.10 |
| $V_M$ ($Å^3$ $Da^{-1}$) | 2.10 | 2.04 | 2.03 | 1.99 | 1.99 |
| Solvent content (%) | 41.42 | 39.79 | 39.53 | 38.36 | 38.12 |
| **Resolution range (Å)** | 29.1-1.83 (1.87-1.83) | 53.51-2.30 (2.38-2.30) | 52.81-1.60 (1.63-1.60) | 28.12-1.60 (1.63-1.60) | 36.78-1.67 (1.69-1.67) |
| Unique reflections | 16704 (978) | 8891 (828) | 25154 (834) | 24583 (845) | 22831 (940) |
| Multiplicity | 5.0 (5.0) | 4.8 (4.7) | 4.6 (2.9) | 4.9 (4.4) | 4.4 (3.8) |
| Completeness (%) | 96.9 (93.6) | 100.0 (100.0) | 96.6 (65.0) | 95.0 (66.6) | 98.9 (79.8) |
| $<I/\sigma(I)>$ | 9.4 (1.9) | 8.4 (2.4) | 21.9 (5.5) | 13.7 (2.9) | 14.9 (2.9) |
| $R_{merge}$ (%) | 9.2 (81.5) | 12.3 (59.2) | 5.4 (13.8) | 6.4 (56.8) | 5.4 (41.8) |
| $CC_{1/2}$ | 0.996 (0.520) | 0.991 (0.611) | 0.998 (0.966) | 0.997 (0.797) | 0.996 (0.716) |
| **Refinement and model statistics** | | | | | |
| $R$ factor (%) | 18.29 | 19.13 | 16.39 | 16.15 | 18.40 |
| $R_{free}$ (%) | 23.11 | 23.74 | 18.1 8 | 18.34 | 19.97 |
| R.M.S Bond lengths (Å) | 0.010 | 0.009 | 0.008 | 0.006 | 0.005 |
| R.M.S Bond angles (°) | 1.104 | 0.999 | 1.012 | 0.949 | 0.860 |
| **Ramachandran plot statistics (residues in %)** | | | | | |
| Favoured region | 99.51 | 99.51 | 100.0 | 100.0 | 98.07 |
| Allowed region | 0.49 | 0.49 | 0.0 | 0.0 | 1.93 |
| Disallowed region | 0.0 | 0.0 | 0.0 | 0.0 | 0.0 |

**Supplementary Table S2C**

**Data collection and refinement statistics of crystal structures of *Msm*UdgX mutants.**

| **Data collection statistics** | **H109Q-DNA** | **H109S/E52N** | **H109S/Q53A** | **H109S/R184A** | **H109S/R184A -DNA** |
|---|---|---|---|---|---|
| PDB ID | 8IIP | 8IIQ | 8IIR | 8IIS | 8IIT |
| Space group | $P2_1$ | $P2_1$ | $P2_1$ | $P2_1$ | $P2_1$ |
| a (Å) | 36.56 | 36.55 | 36.5 | 36.36 | 36.44 |
| b (Å) | 51.34 | 51.46 | 51.15 | 51.65 | 51.42 |
| c (Å) | 54.88 | 54.69 | 54.96 | 54.58 | 54.94 |
| β (°) | 104.67 | 104.92 | 104.42 | 105.00 | 104.84 |
| $V_M$ ($Å^3$ $Da^{-1}$) | 2.03 | 1.99 | 2.10 | 2.02 | 1.99 |
| Solvent content (%) | 39.56 | 38.17 | 41.55 | 39.16 | 38.24 |
| **Resolution range (Å)** | 28.13-1.63 (1.66-1.63) | 36.87-2.10 (2.16-2.10) | 53.23-2.28 (2.37-2.28) | 28.11-1.60 (1.63-1.60) | 26.55-1.60 (1.63-1.60) |
| Unique reflections | 24686 (1270) | 10962 (936) | 9050 (925) | 24280 (831) | 25385 (908) |
| Multiplicity | 4.7 (4.5) | 3.7 (3.6) | 3.0 (3.0) | 4.9 (4.2) | 4.4 (3.8) |
| Completeness (%) | 100.0 (100.0) | 94.6 (100) | 99.9 (99.9) | 94.3 (66.2) | 98.1 (71.8) |
| $<I/\sigma(I)>$ | 12.7 (2.5) | 5.5 (2.6) | 6.2 (2.2) | 28.2 (16.8) | 18.5 (6.0) |
| $R_{merge}$ (%) | 6.8 (52.0) | 13.2 (37.1) | 9.8 (31.5) | 4.0 (5.9) | 4.7 (27.6) |
| $CC_{1/2}$ | 0.996 (0.684) | 0.979 (0.811) | 0.986 (0.822) | 0.996 (0.985) | 0.995 (0.910) |
| **Refinement and model statistics** | | | | | |
| *R* factor (%) | 17.56 | 18.60 | 17.89 | 15.29 | 15.58 |
| $R_{free}$ (%) | 20.27 | 24.17 | 23.81 | 17.03 | 18.04 |
| R.M.S Bond lengths (Å) | 0.006 | 0.009 | 0.003 | 0.019 | 0.019 |
| R.M.S Bond angles (°) | 0.925 | 0.967 | 0.551 | 1.746 | 1.685 |
| **Ramachandran plot statistics (residues in %)** | | | | | |
| Favoured region | 100.0 | 99.50 | 98.53 | 99.53 | 100.0 |
| Allowed region | 0.0 | 0.50 | 1.47 | 0.47 | 0.0 |
| Disallowed region | 0.0 | 0.0 | 0.0 | 0.0 | 0.0 |

**Supplementary Table S3**

Active site cavity volume of *Msm*UdgX H109 mutants. Active site cavity volume was measured by calculating the volume of 6 Å radius sphere from CD of E52 residue, with a grid space of 0.1 Å and volume space of 0.2 Å

| Protein | Atoms in input pdb | Grid (Å) | Grid Width (Å) | Volume (Å$^3$) |
|---|---|---|---|---|
| **WT** | 14688 | 98 x 98 x 98 | 19.60 | 582.9 |
| **H109K** | 14610 | 100 x 100 x 100 | 20.00 | 554.6 |
| **H109Q** | 15793 | 100 x 100 x 100 | 20.00 | 597.0 |
| **H109G** | 15502 | 102 x 102 x 102 | 20.40 | 609.3 |
| **H109S** | 17103 | 102 x 102 x 102 | 20.40 | 643.9 |
| **H109C** | 17633 | 100 x 100 x 100 | 20.00 | 663.1 |
| **H109A** | 18283 | 102 x 102 x 102 | 20.40 | 683.1 |

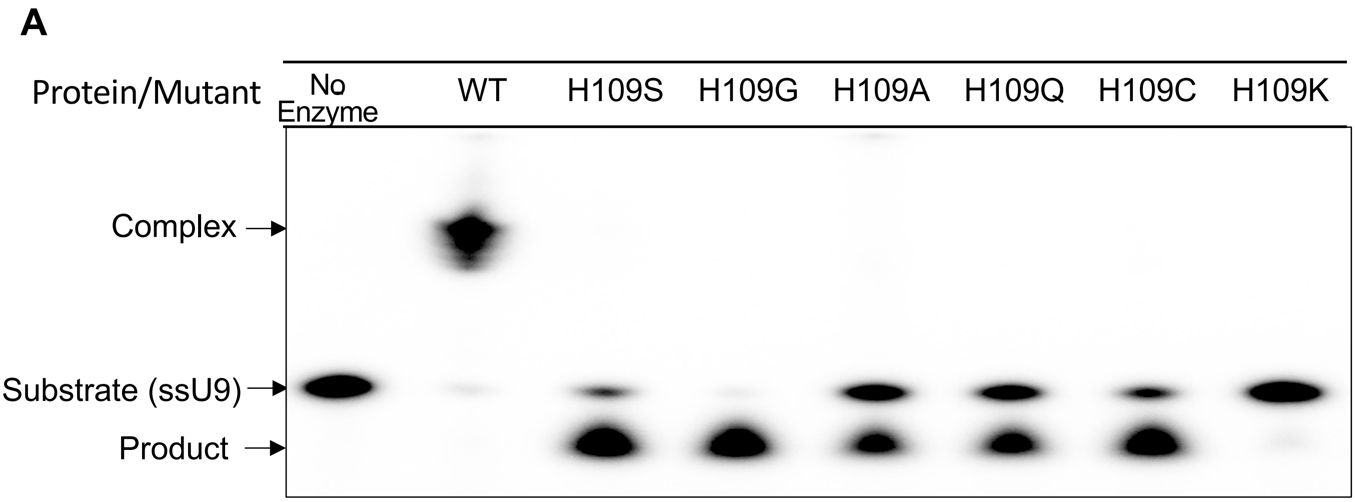


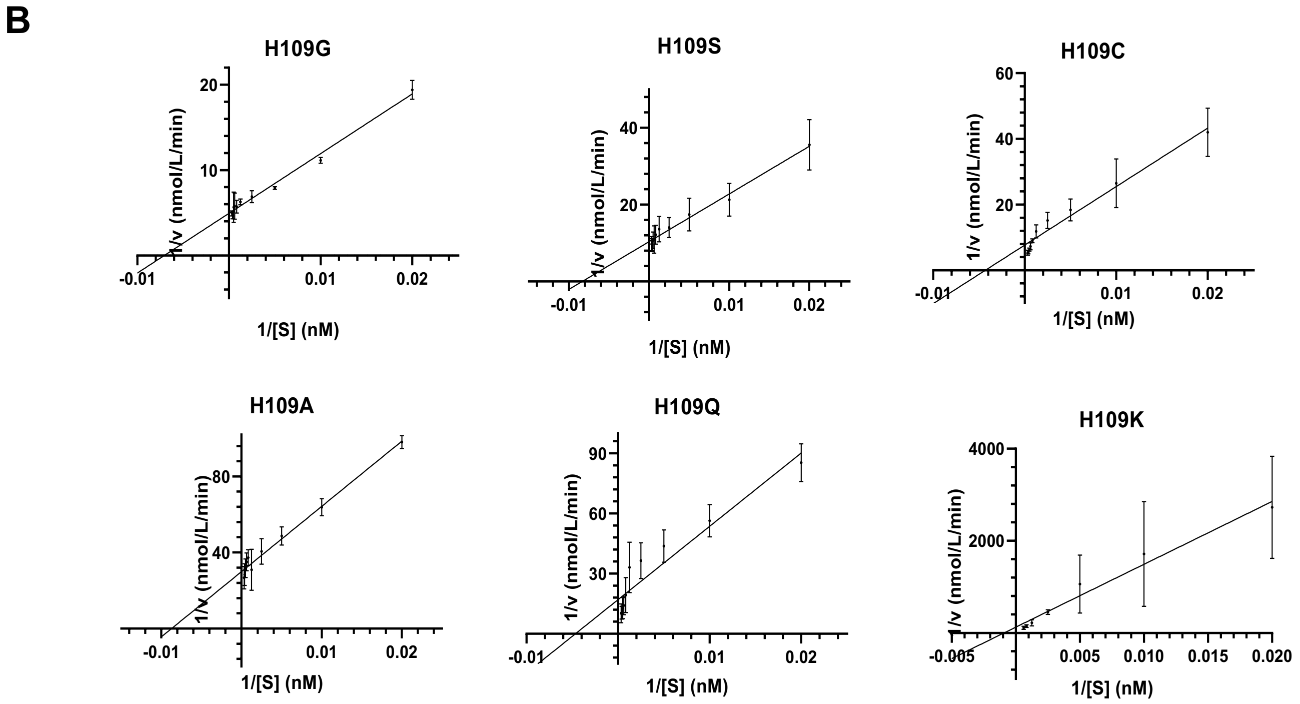


**Supplementary Figure S1: A**. Activity assays of *Msm*UdgX H109 mutants performed using ssU9 substrates labelled with $^{32}P$ at the 5′ end. The reaction was carried out for 20 min, resolved on 8M urea PAGE and analysed by phosphorimaging. **B**. Lineweaver Burk (LB) plots of *Msm*UdgX H109 mutants. Rate of the reaction at each substrate concentration was calculated from the slopes of the rates obtained at 2, 4, 6 and 8 min of reaction. Inverse of substrate concentration was plotted on X-axis and inverse of rate was plotted on Y-axis.

Fig. S2

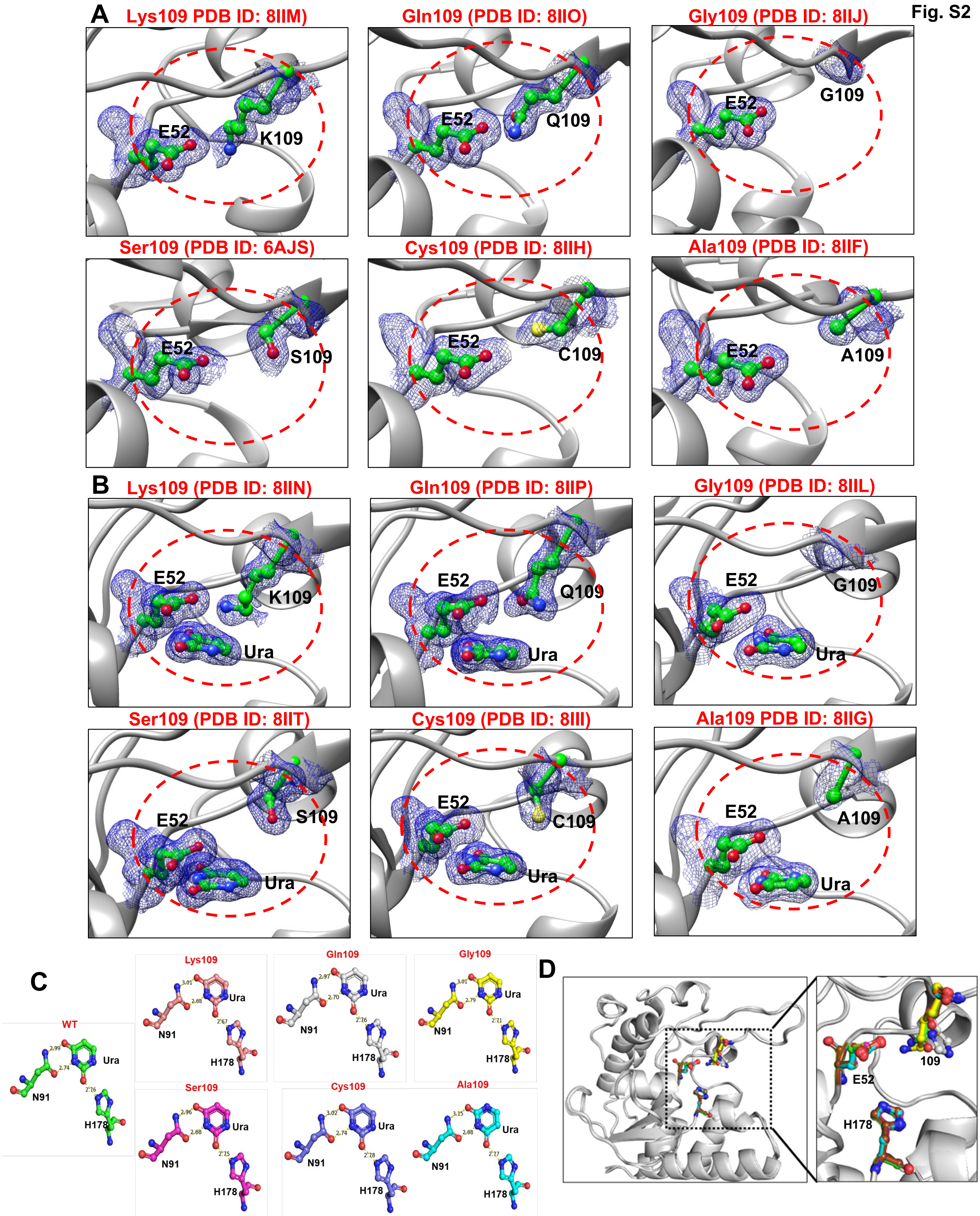


**Supplementary Figure S2: A**. Crystal structures of H109 mutants. E52 and H109 mutants are shown in atomic colours. *2mF$_o$-DF$_c$* map is contoured at 0.5 σ and presented as blue mesh. **B**. Crystal structures of H109 mutants with TTUTT substrate. E52, mutations at H109 and uracil are shown in atomic colors. *2mF$_o$-DF$_c$* map is contoured at 0.5 σ and presented as blue mesh. **C.** Hydrogen bonding distances between O2 of Ura and NE2 of H178, N3 of Ura and OD of N91, O4 of Ura and ND of N91 in *Msm*UdgX and its H109 mutants. The bond distances are represented in Å units. **D.** Structural rationale for the effect of H109 mutations on the positioning of E52 and H178 in *Msm*UdgX. LSQ superposed images of *Msm*UdgX and its H109 mutants [H109K (yellow), H109Q (green), H109G (cyan), H109S/R184A (orange), H109C (magenta), and H109A (brown)]. The positions of E52, H109 mutants, and H178 are highlighted as balls and sticks with the respective colors of the mutants.

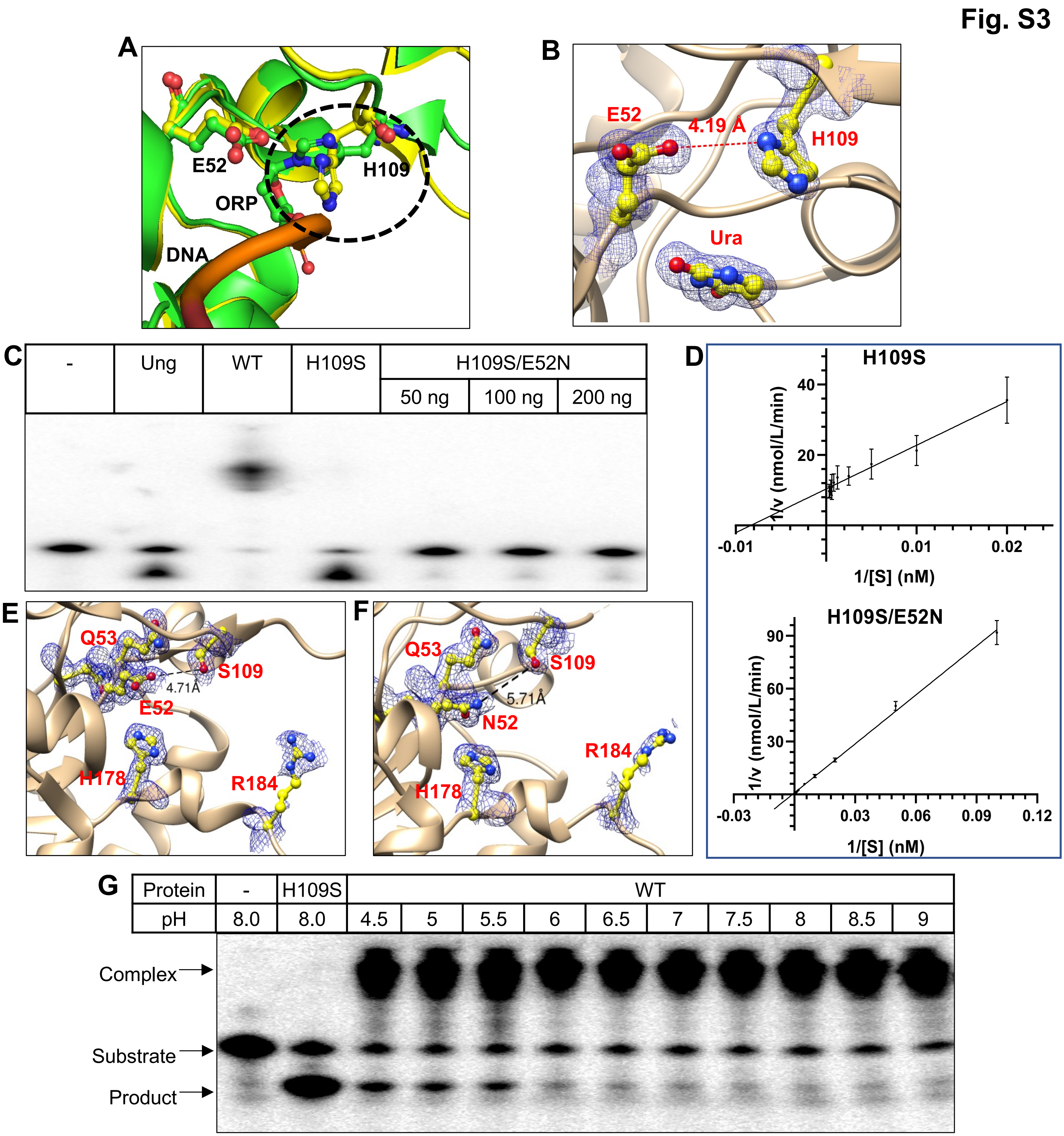


**Supplementary Figure S3: A**. Snapshot showing the position of H109 in *Msm*UdgX (Re-refined) (yellow) (PDB ID: 6AIL) and DNA bound form (green) (PDB ID: 6AJO). **B**. Crystal structure of *Msm*UdgX with uracil in its active site, (post excision) (PDB ID: 8IIE). Dashed line indicates the distance between OE2 of E52 and ND1 of H109. $2mF_o\text{-}DF_c$ map is contoured at 0.5 σ and presented as blue mesh. **C**. The activity assays of *Msm*UdgX mutants performed using ssU9 substrates labelled with $^{32}$P at the 5´ end. The reactions were carried out for 20 min, resolved on 8M urea PAGE and analysed by phosphorimaging. **D**. Lineweaver Burk (LB) plot of *Msm*UdgX H109S and, H109S/E52N mutants. Rate of the reaction at each substrate concentration was calculated from the slope of the rates obtained at 2, 4, 6 and 8 min of reaction. Inverse of substrate concentration was plotted on X-axis and inverse of rate was plotted on Y-axis for the LB plot. **E** and **F**. Snapshots of crystal structure of *Msm*UdgX H109S (PDB ID: 6AJS) and H109S/E52N (PDB ID: 8IIQ) mutants. E52 (**E**), N52 (**F**), S109, H178, R184 and Q53 are shown in atomic colours. $2mF_o\text{-}DF_c$ map is contoured at 0.5 σ and presented as blue mesh. Hydrogen bond between OG of S109 and ND2 of N52 or OE2 of E52 are indicated by dashed lines. **G**. Activity of *Msm*UdgX from pH 4.5-9.0. Activity assays at varying pH was performed using ssU9 substrates labelled with $^{32}$P at the 5´ end. The reaction were carried out for 20 min, resolved on 8M urea PAGE and analysed by phosphorimaging.

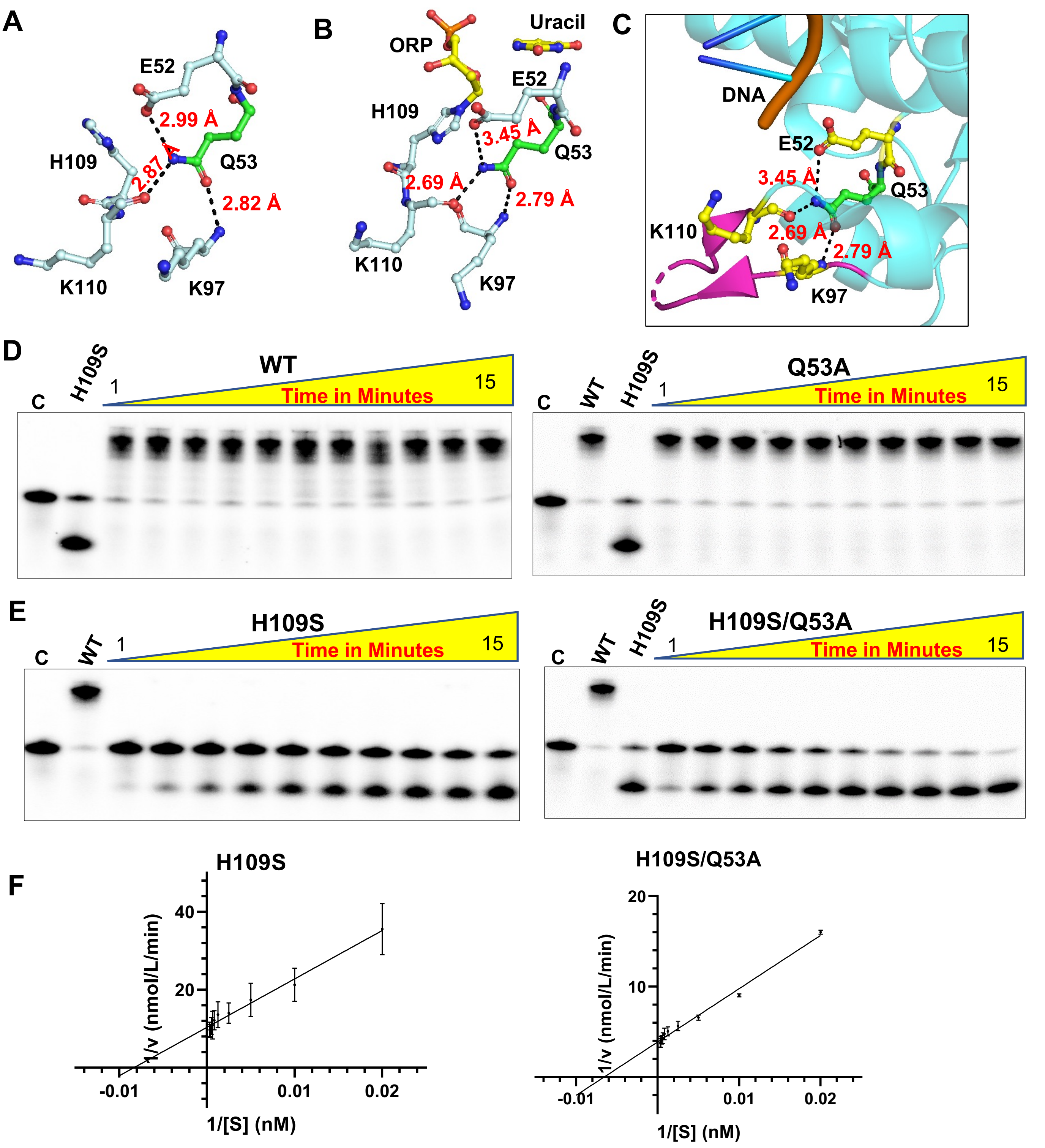


**Supplementary Figure S4:** **A**. Ligplot⁺ image showing the hydrogen bonds made by Q53 with K97, K110, and E52 in *Msm*UdgX (PDB ID: 6AIL). Hydrogen bonds are shown in dashed lines. **B**. Ligplot⁺ image showing the hydrogen bonds made by Q53 with K97, K110, and E52 in *Msm*UdgX in complex with AP DNA (PDB ID: 6AJO). **C.** Crystal structure showing the interaction between K97, Q53 and K110 in the DNA bound UdgX (PDB ID: 6AJO). **D** and **E** **(Figs for WT and H109S have been taken from Ahn et. al)**. Time course of activity analysis of *Msm*UdgX and its mutants. The activity assays were carried out with ssU9 DNA substrate at 37 °C for 15 min. The reaction times for lanes 3 to 13 in *Msm*UdgX correspond to 1, 2, 3, 4, 5, 6, 7, 8, 9, 10 and 15 min, respectively. The reaction times for lanes 3 to 12 in H109S corresponds to 1, 2, 3, 4, 5, 6, 7, 8, 9 and 15 min, respectively. The reaction times for lanes 4 to 13 in Q53A and, H109S/Q53A correspond to 1, 2, 3, 4, 5, 6, 7, 8, 9 and 15 min, respectively. The reactions were resolved on 8M urea-PAGE (15%) and analyzed by phosphorimaging. **F**. Lineweaver Burk (LB) plot of *Msm*UdgX H109S and H109S/Q53A mutants kinetic assay. Rate of the reaction at each substrate concentration was calculated from the slope of rates obtained at 2, 4, 6 and 8 minutes of reaction. Inverse of substrate concentration was plotted on X-axis and inverse of rate was plotted on Y-axis.

**Fig. S5**

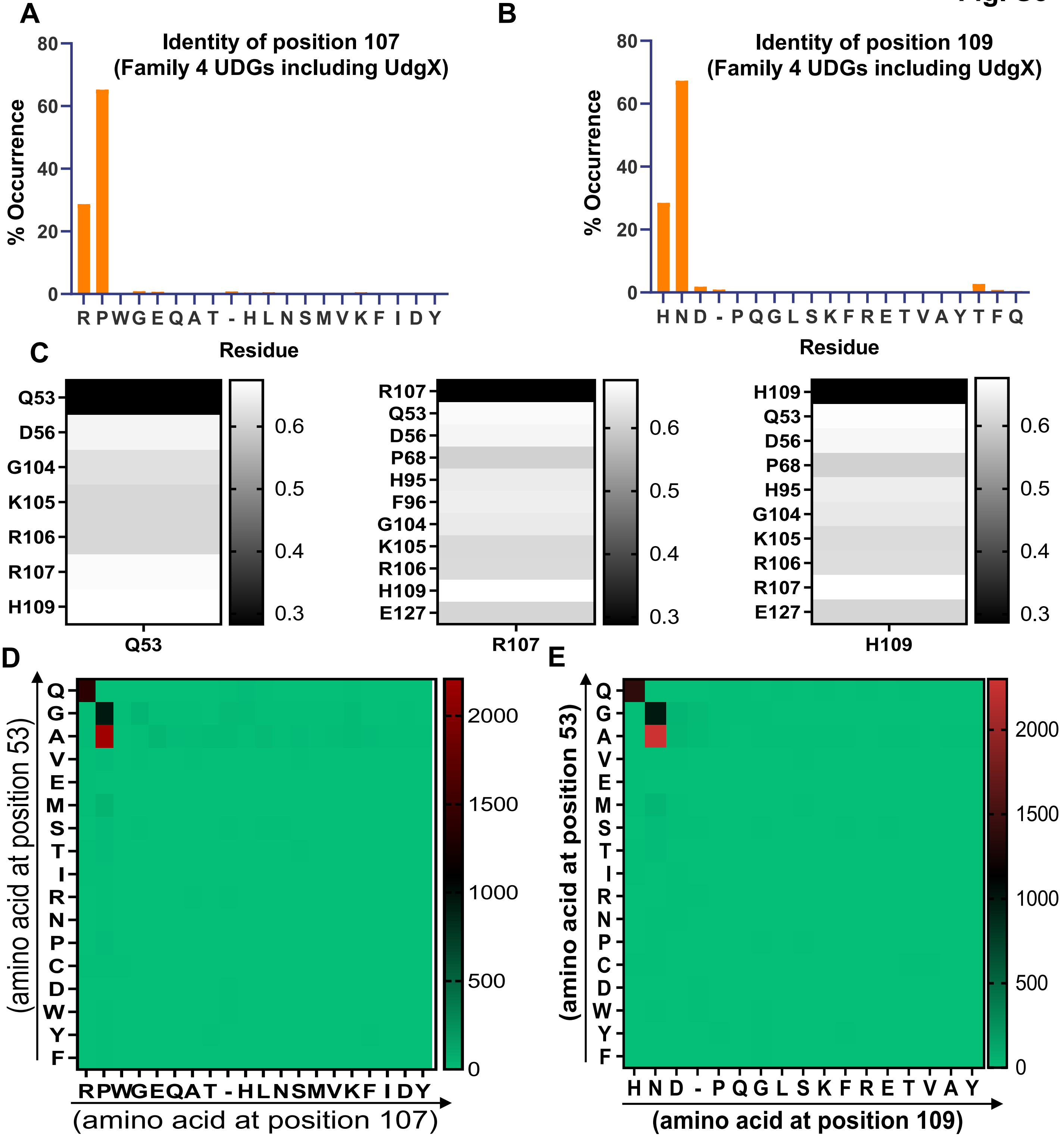


**Supplementary Figure S5: A and B**. XY graph depicting the percent occurrences of amino acids at positions 107 **(A)** and 109 **(B)** in family 4 UDGs (including UdgXs). 5000 individual sequences were aligned using default parameters of ClustalW (without any gaps) to obtain the multiple sequence alignment (MSA) file. Percent occurrences were calculated by dividing number of appearances of individual residue by total number of samples (5000). **C**. Nearest coevolving residues for Q53, R107 and H109. Q53, R107 and H109 are as shown. **D and E**. Cooccurrences of amino acids at position 53 with respect to the amino acids at positions 107 **(D)**, and 109 **(E)**. Data were obtained by keeping the equivalents of positions 107 or 109 fixed and identifying the corresponding amino acids at position 53 (Note: In panel D, when R occurs at position 107 (X-axis), the amino acid at position 53 is almost exclusively Q (Y-axis). Our analysis shows that all of these sequences correspond to UdgX. The other predominant amino acid at position 107 is P, with the corresponding amino acids as A or G at position 53, and all of these proteins were family 4 UDGs excluding UdgX. Any other co-occurrences were insignificant (<2%). Similarly, in panel E, when H occurs at position 109 (X-axis), the amino acid at position 53 is almost exclusively Q (Y-axis). Our analysis shows that all of these sequences correspond to UdgX. The other predominant amino acid at position 109 is N, and its counterparts at position 53 ar A or G, and all of these proteins were family 4 UDGs excluding UdgX. Any other co-occurrences were insignificant (<2%).

**Fig. S6**

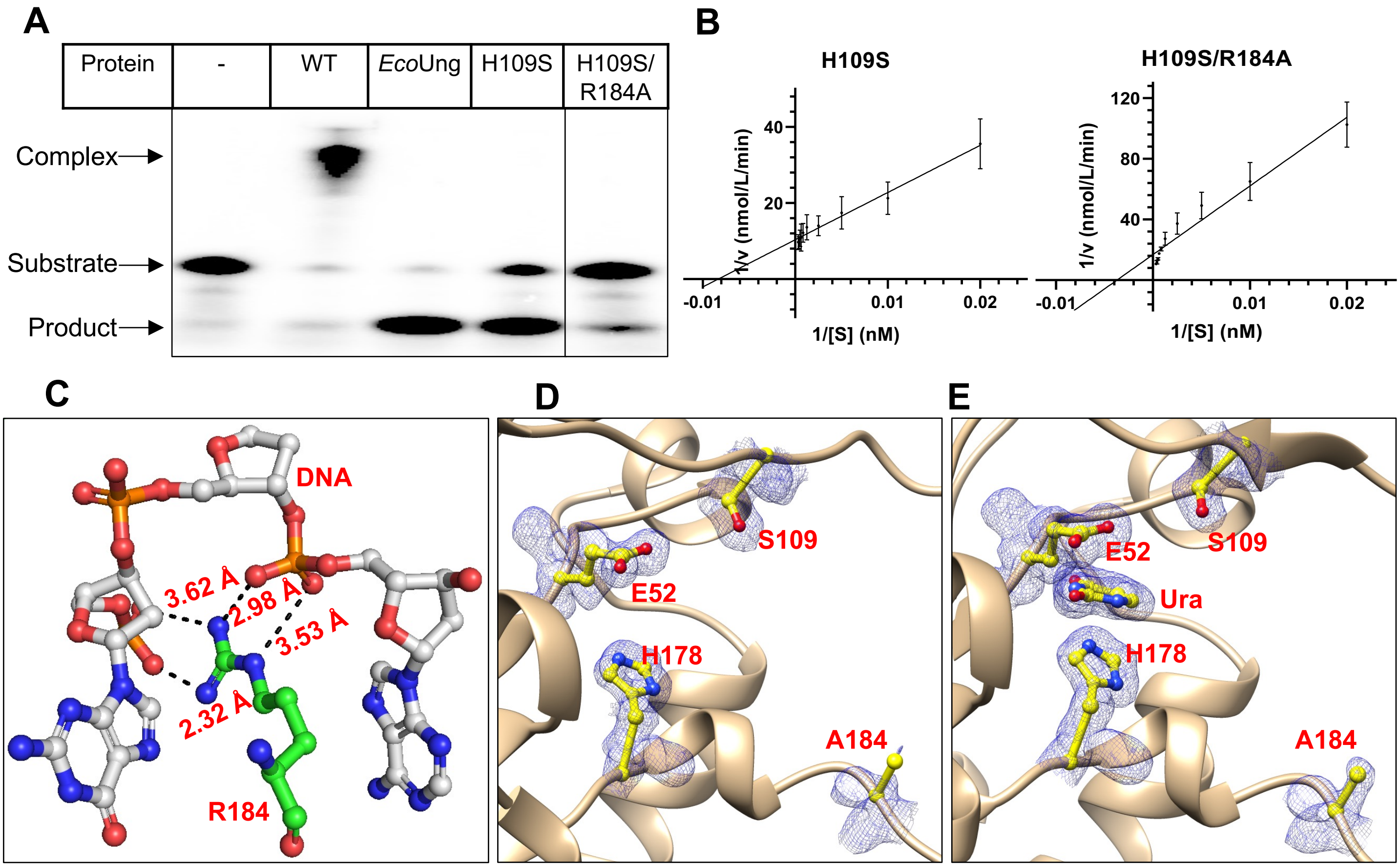


**Supplementary Figure S6:** **A**. Activity assays of *Msm*UdgX mutants performed using ssU9 substrates labelled with $^{32}P$ at the 5′ end. The reaction was carried out for 20 min, resolved on 8M urea PAGE and analysed by phosphorimaging. **B**. Lineweaver Burk (LB) plot of *Msm*UdgX H109S and H109S/R184A mutants. Rate of the reaction at each substrate concentration was calculated by taking the slope of the rates obtained at 2, 4, 6 and 8 min of reaction. Inverse of substrate concentration was plotted on X-axis and inverse of rates were plotted on Y-axis. **C**. Hydrogen bonding interactions by R184 with OP1 and OP2 of (P+1) and (P-1) of DNA backbone (crystal structure from Tu. et. al. 2019). **D and E**. Snapshots of crystal structure of *Msm*UdgX H109S/R184A (PDB ID: 8IIS) and H109S/R184A-uracil (PDB ID: 8IIT) (crystalised with TTUTT). E52, S109, H178, and R184 are shown in atomic colors. $2mF_o$-$DF_c$ map is contoured at 0.5 σ and presented as blue mesh.
